# Esaxerenone Inhibits Renal Angiogenesis and Endothelial-Mesenchymal Transition via the VEGFA and TGF-β1 Pathways in Aldosterone-Infused Mice

**DOI:** 10.3390/ijms241411766

**Published:** 2023-07-21

**Authors:** Xiaomeng Gao, Jingyue Chang, Yi Chang, Lili Fan, Ziqian Liu, Cuijuan Zhang, Tatsuo Shimosawa, Fan Yang, Qingyou Xu

**Affiliations:** 1 Graduate School, Hebei University of Chinese Medicine, Shijiazhuang 050200, China; 2 Hebei Key Laboratory of Integrative Medicine on Liver-Kidney Patterns, Hebei University of Chinese Medicine, Shijiazhuang 050200, China; 3 Institute of Integrative Medicine, College of Integrative Medicine, Hebei University of Chinese Medicine, Shijiazhuang 050200, China; 4 Department of Clinical Laboratory, School of Medicine, International University of Health and Welfare, Narita 286-8686, Japan

**Keywords:** renal fibrosis, aldosterone, mineralocorticoid receptor blocker, angiogenesis, VEGFA

## Abstract

Renal fibrosis is an inevitable process in the progression of chronic kidney disease (CKD). Angiogenesis plays an important role in this process. Vascular endothelial cells are involved in renal fibrosis by phenotypic transformation and secretion of extracellular matrix. Aldosterone stimulates mineralocorticoid receptor (MR) activation and induces inflammation, which is important for angiogenesis. Clinically, MR blockers (MRBs) have a protective effect on damaged kidneys, which may be associated with inhibition of angiogenesis. In this study, we used aldosterone-infused mice and found that aldosterone induced angiogenesis and that endothelial-mesenchymal transition (EndMT) in neovascular endothelial cells was involved in renal fibrosis. Notably, aldosterone induced inflammation and stimulated macrophages to secrete vascular endothelial growth factor (VEGF) A to regulate angiogenesis by activating MR, whereas EndMT occurred in response to transforming growth factor-β1 (TGF-β1) induction and participated in renal fibrosis. These effects were antagonized by the MRB esaxerenone. These findings suggest that reducing angiogenesis may be an effective strategy for treating renal fibrosis.

## 1. Introduction

Chronic kidney disease (CKD) is considered a global public health problem, resulting in health issues and financial burdens due to its increasing incidence and prevalence worldwide, especially among elderly populations [1]. Renal fibrosis is the ultimate common pathological manifestation of CKD due to various causes, and it is also the common pathway from CKD to end-stage renal failure. Renal fibrosis, which is characterized by glomerulosclerosis, tubular atrophy, and interstitial fibrosis, is a manifestation of chronic and persistent renal injury [2].

Angiogenesis plays a pivotal role in many pathological processes, including CKD [3]. Inflammatory mediators and hypoxia are the most important factors that induce angiogenesis, and both can be induced by aldosterone [4,5,6]. Endothelial–mesenchymal transition (EndMT) occurs during angiogenesis and participates in the progression of organ fibrosis. The protective effect of mineralocorticoid receptor blockers (MRBs) on organ fibrosis may be related to the inhibition of angiogenesis [7].

In this study, we focused on the role of aldosterone in renal angiogenesis and EndMT in mice, as well as the inhibitory effect of the MRB esaxerenone, to explore the occurrence and treatment of renal fibrosis. There are three questions that need to be answered. (1) Can aldosterone induce angiogenesis? (2) Can aldosterone induce EndMT? (3) How does aldosterone work? Here, we used a 12-week aldosterone-infused mouse model and in vitro cell culture to observe the effects of aldosterone, providing new insights into the mechanisms of aldosterone-induced chronic renal fibrosis.

## 2. Results

### 2.1. Esaxerenone Reverses Renal Injury in Aldosterone-Treated Mice

**Figure 1:**
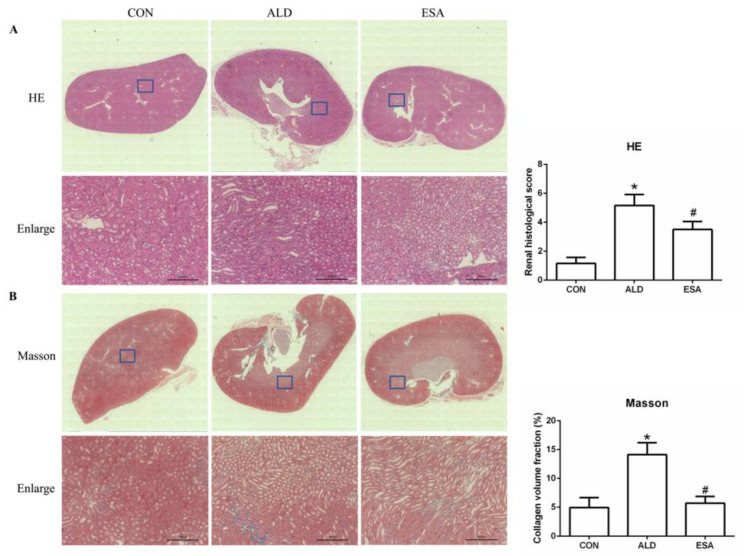

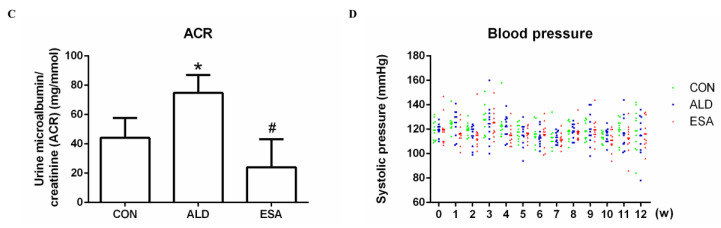
Kidney sections were stained with H&E to examine morphological changes and inflammatory cell infiltration (**A**) and Masson’s trichrome was used to examine fibrosis (**B**) (n = 6), scale bar = 200 µm. (**C**) The ACR was evaluated to determine renal function (n = 6). (**D**) Measurement of SBP (n = 10). The data are presented as the mean ± SD, * *p* < 0.05 compared with the CON group. ^#^ *p* < 0.05 compared with the ALD group.

Aldosterone-induced organ damage is associated with inflammation. We confirmed these changes after continuous infusion of aldosterone in mice with osmotic pumps. As shown in Figure 1A, inflammatory cell infiltration was increased, and tubular damage was aggravated in the aldosterone group (ALD). Masson staining showed that the amount of collagen deposition in the ALD group was higher than that in the control group (CON) (Figure 1B). Urine creatinine (UCr) and microalbuminuria (mAlb) were measured, and the mAlb/UCr ratio (ACR) was calculated to detect kidney function in mice. Consistent with the pathological observations, ACR levels revealed impaired renal function in the ALD group compared with the CON group (Figure 1C). In the esaxerenone treatment (ESA) group, these effects were suppressed by the specific MRB (Figure 1A–C). In addition, we found no significant difference in blood pressure levels among the groups of mice (Figure 1D).

### 2.2. Esaxerenone Inhibits Renal Angiogenesis in Aldosterone-Treated Mice

**Figure 2:**
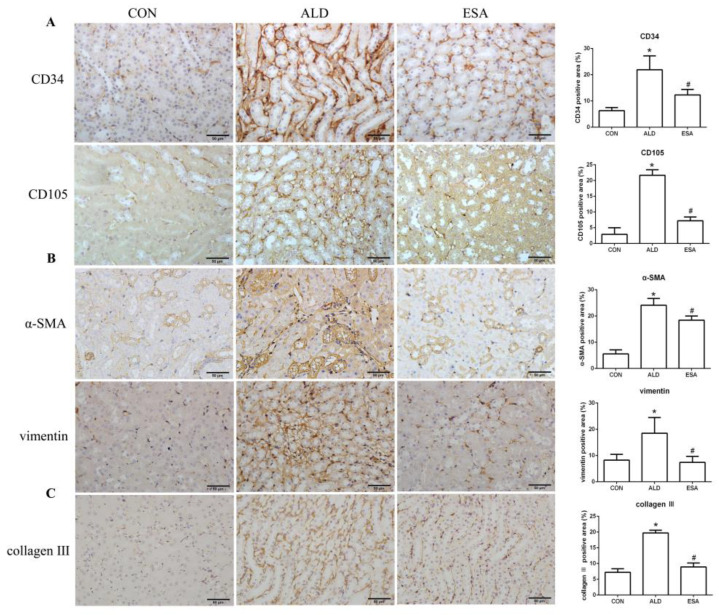

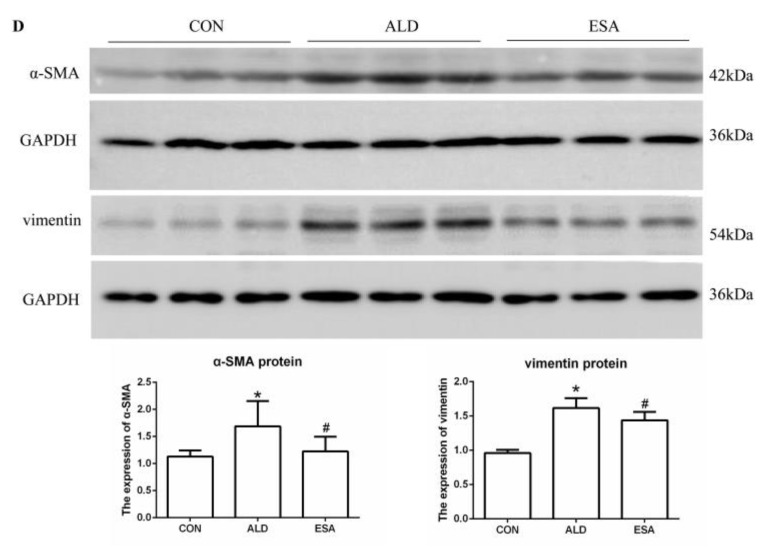
ESA inhibits renal angiogenesis in ALD-treated mice. (**A**) Immunohistochemical staining using antibodies against CD34 and CD105 to examine angiogenesis (n = 6). (**B**) Immunohistochemical staining using antibodies against α-SMA and vimentin to examine renal infiltration of myofibroblasts (n = 6). (**C**) Immunohistochemical staining for collagen III (n = 6). (**D**) Western blot analysis of α-SMA and vimentin in the kidneys (n = 6). The data are presented as the mean ± SD, * *p* < 0.05 compared with the CON group. ^#^ *p* < 0.05 compared with the ALD group. Scale bar = 50 µm.

CD34 and CD105 have been used to identify vascular endothelial cells and new vascular endothelial cells. Angiogenesis was significantly observed in the renal interstitium of aldosterone-infused mice but was inhibited by esaxerenone (Figure 2A). Myofibroblasts were evaluated by immunohistochemical staining with antibodies against α-SMA and vimentin, which are specific markers of myofibroblasts. Myofibroblasts accumulated in the renal interstitium in the kidneys of aldosterone-treated mice, and this accumulation was reversed by esaxerenone treatment (Figure 2B). Immunostaining of collagen III supported this conclusion; there was more collagen in the kidneys of the aldosterone-treated mice than those of the CON and ESA groups (Figure 2C). Likewise, Western blot analysis of kidney tissue proteins extracted in vivo (Figure 2D) further confirmed these results.

### 2.3. Esaxerenone Alleviates the Secretion of Vascular Endothelial Growth Factor A (VEGFA) by Macrophages in Aldosterone-Treated Mice

**Figure 3:**
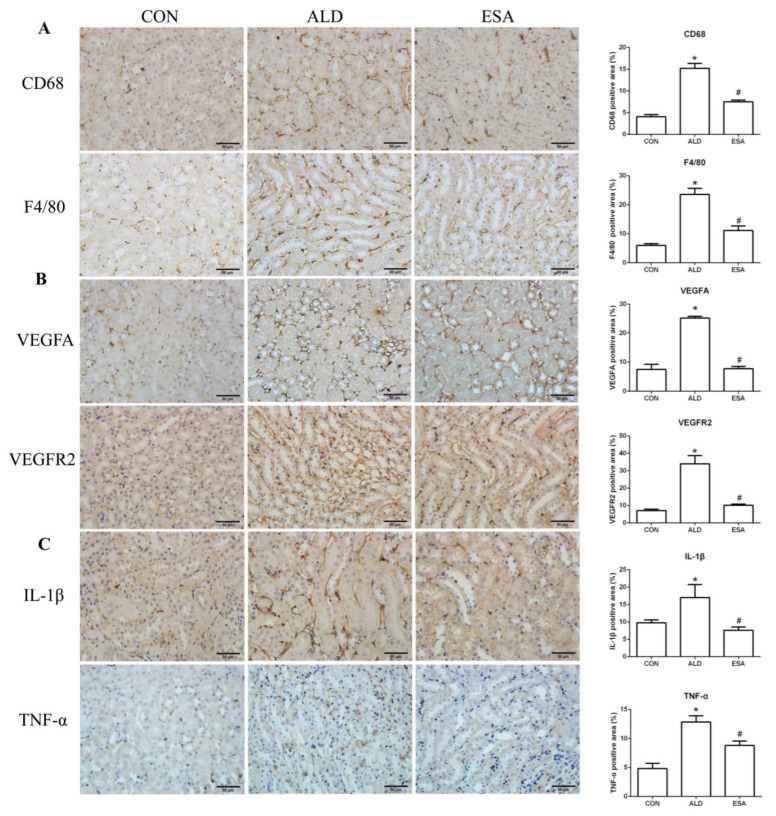

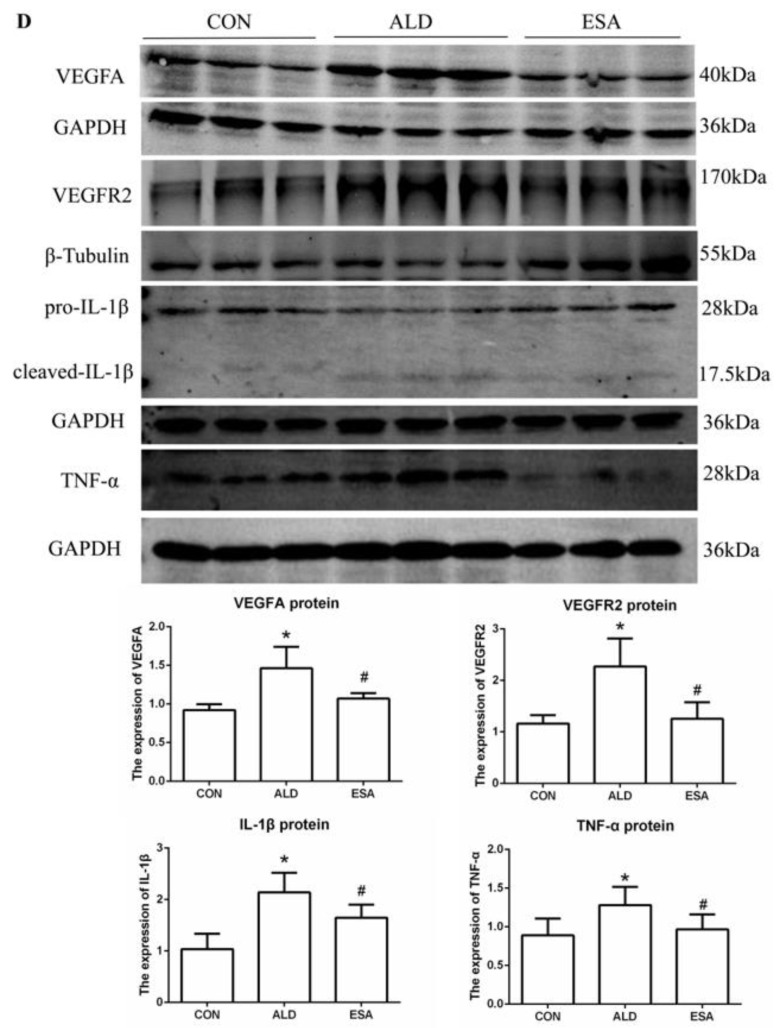

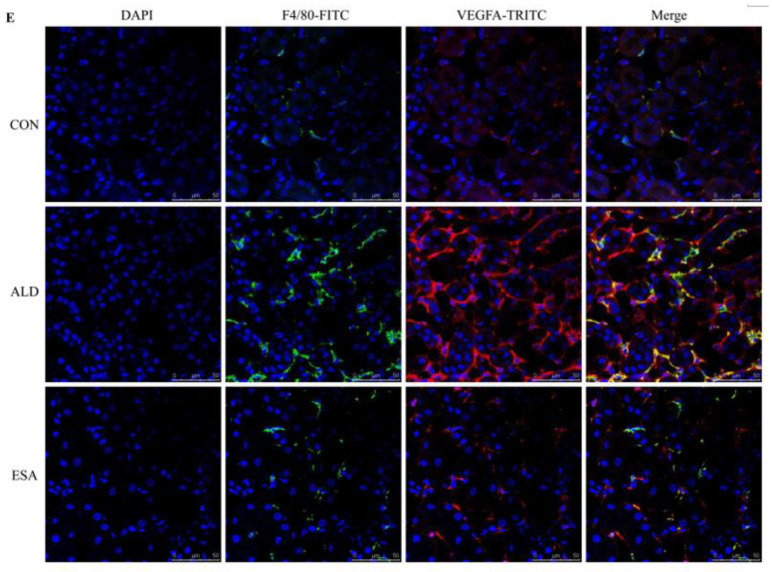
ESA antagonized the increased infiltration of macrophages. (**A**) Immunohistochemical staining using antibodies against CD68 and F4/80 to examine renal infiltration of macrophages (n = 6). (**B**) Immunohistochemical staining using antibodies against VEGFA and VEGFR2 to examine proangiogenic factors (n = 6). (**C**) Immunohistochemical staining using antibodies against IL-1β and TNF-α to examine the formation of an inflammatory environment (n = 6). (**D**) Western blot analysis of VEGFA, VEGFR2, IL-1β, and TNF-α in kidneys (n = 6). (**E**) Immunofluorescence staining of kidney sections with antibodies against the macrophage marker F4/80 (FITC, green) and VEGFA (TRITC, red) to show that VEGFA was mainly derived from macrophages (cells coexpressing both markers); nuclei were stained with DAPI (blue). The data are presented as the mean ± SD, * *p* < 0.05 compared with the CON group. ^#^ *p* < 0.05 compared with the ALD group. Scale bar = 50 µm.

F4/80 and CD68 were used to identify macrophages, and obvious macrophage infiltration was observed in the renal interstitium of the aldosterone-treated mice, which was also a specific manifestation of aldosterone-induced inflammation, and the increase in infiltrating macrophages was inhibited by esaxerenone (Figure 3A). VEGFA is, typically, secreted by renal tubular epithelial cells and macrophages in the kidney [8,9]. This factor plays a key role in angiogenesis by interacting with vascular endothelial growth factor receptor 2 (VEGFR2) to regulate vascular permeability and production [10]. First, we performed an immunohistochemical analysis by staining kidney sections with VEGFA and VEGFR2 and found that their expression in the ALD group was higher than that in the CON group, and this effect was reduced in the ESA group (Figure 3B). In addition, we detected renal protein expression of VEGFA and VEGFR2, which was consistent with the previous results (Figure 3D). Then, to verify whether aldosterone stimulates macrophages to secrete VEGFA, we performed immunofluorescence staining of kidney sections with F4/80 and VEGFA. As shown in Figure 3E, the expression level of F4/80 was significantly upregulated in the aldosterone-treated mice, which was consistent with the immunohistochemistry results (Figure 1A). VEGFA also showed high expression, and most of the VEGFA-expressing regions were positive for F4/80 and were highly overlapping in the renal interstitium. Moreover, we found that VEGFA is secreted mainly by macrophages in the kidneys of the aldosterone-treated mice.

Macrophages are pleiotropic inflammatory cells that participate in inflammatory reactions, so we measured the expression of interleukin-1β (IL-1β) and tumor necrosis factor-α (TNF-α) by immunohistochemistry. In the aldosterone-treated mice, the expression levels of IL-1β and TNF-α were increased, suggesting that aldosterone may promote angiogenesis by inducing inflammation (Figure 3C). In addition, quantitative analysis of the Western blot results was similar to that of immunohistochemistry (Figure 3D).

### 2.4. Esaxerenone Alleviates EndMT in the Kidneys of Aldosterone-Treated Mice

**Figure 4:**
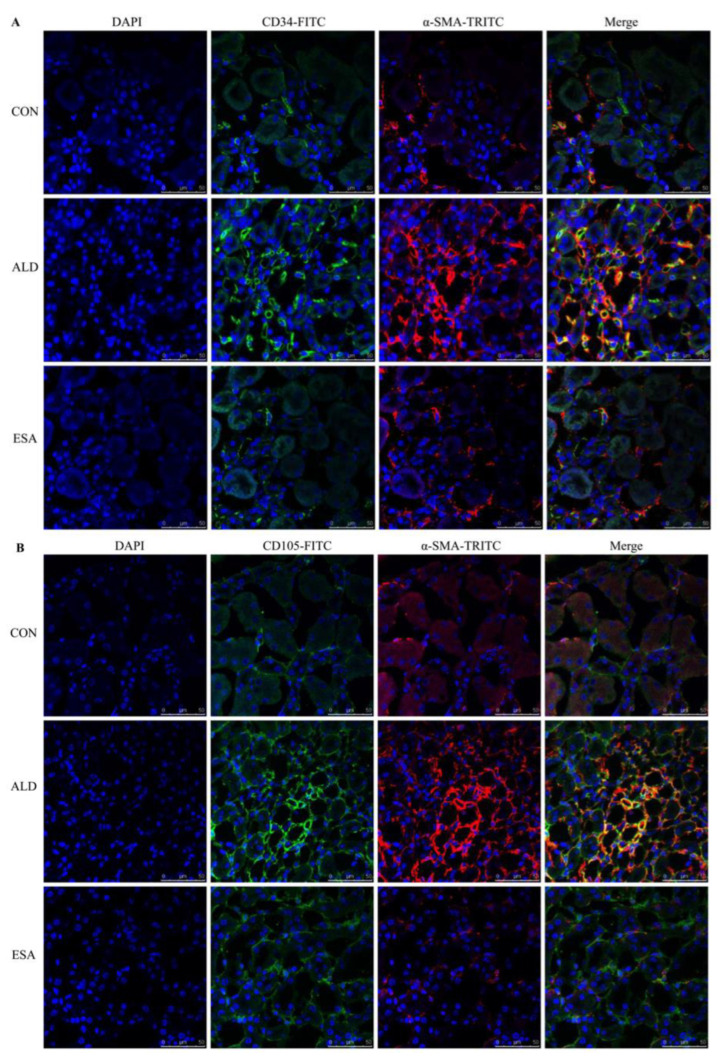

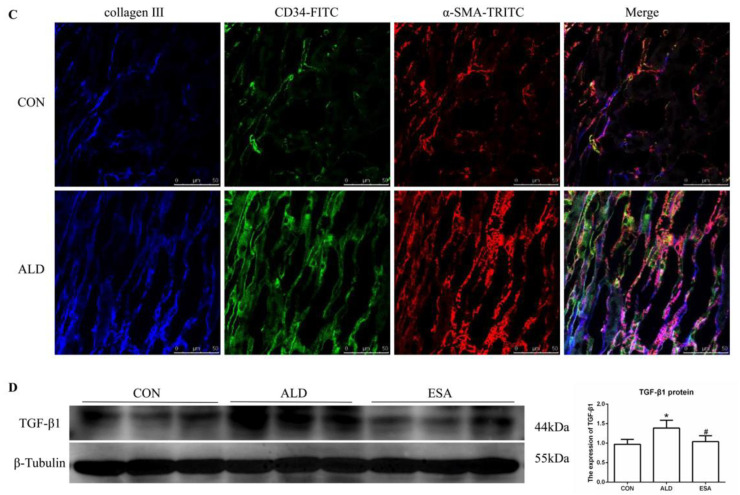
EndMT in ALD-infused mice. (**A**) Immunofluorescence staining of kidney sections with antibodies against CD34 (FITC, green) and the myofibroblast marker α-SMA (TRITC, red) to identify EndMT (cells coexpressing both markers indicate EndMT; nuclei were stained with DAPI (blue)). (**B**) Immunofluorescence staining of kidney sections with antibodies against CD105 (FITC, green) and the myofibroblast marker α-SMA (TRITC, red) to identify EndMT (cells coexpressing both markers indicate EndMT; nuclei were stained with DAPI (blue)). (**C**) Immunofluorescence staining of kidney sections with antibodies against CD34 (green), α-SMA (red), and collagen III (blue). (**D**) Western blot analysis of TGF-β1 in kidneys (n = 6). The data are presented as the mean ± SD, * *p* < 0.05 compared with the CON group. ^#^ *p* < 0.05 compared with the ALD group. Scale bar = 50 µm.

EndMT was evaluated by costaining the kidney with antibodies against α-SMA and the vascular endothelial cell markers CD34 and CD105. As expected, the number of α-SMA and CD34 double-positive cells in the ALD group was higher than that in the CON group, and this effect was attenuated in the ESA group (Figure 4A, Video S1). The costaining results of CD105 and α-SMA were similar to those of CD34 (Figure 4B). In addition, we costained CD34 and α-SMA with collagen III and found that cells expressing CD34 and α-SMA expressed collagen III, and the number of CD34 and α-SMA with collagen III triple-positive cells in the ALD group was higher than that in the CON group, indicating that phenotypically transformed vascular endothelial cells secreted collagen components and participated in fibrosis (Figure 4C). Subsequently, we found that the expression of transforming growth factor-β1 (TGF-β1), one of the downstream molecules of MR, in the ALD group was higher than that in the CON group and decreased in the ESA group (Figure 4D).

### 2.5. Esaxerenone Inhibits Angiogenesis by Inhibiting the Secretion of VEGFA from Macrophages In Vitro

**Figure 5:**
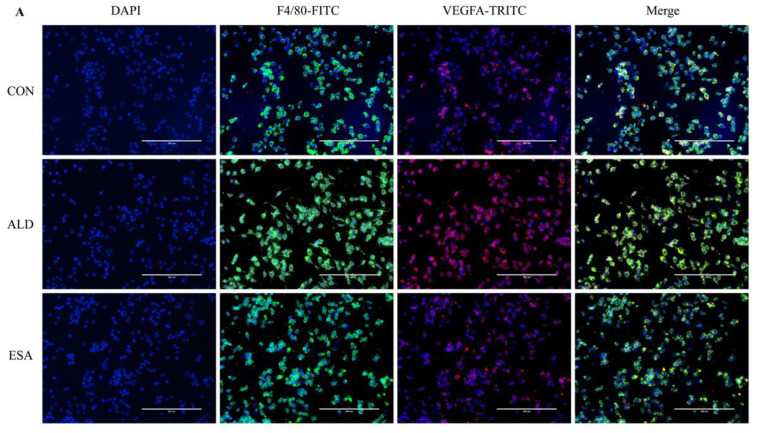

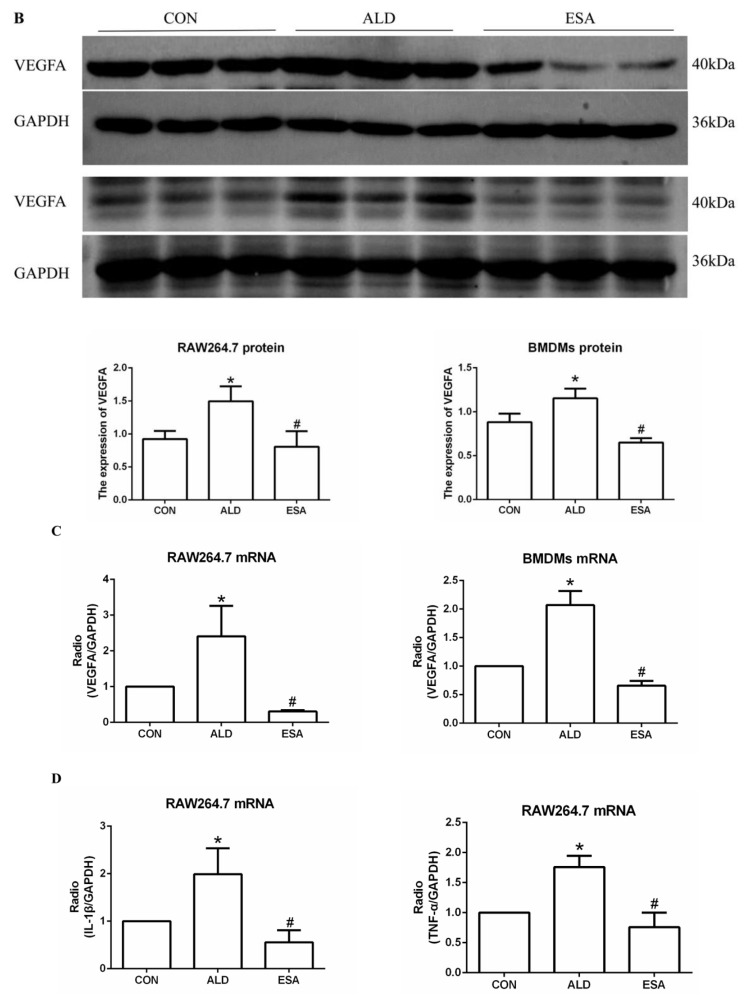
ESA alleviates the secretion of VEGFA by ALD-induced macrophages in vitro. (**A**) Immunofluorescence staining of RAW264.7 cells with antibodies against the macrophage marker F4/80 (FITC, green) and VEGFA (TRITC, red); nuclei were stained with DAPI (blue) (n = 6). (**B**) Western blot analysis of VEGFA in RAW264.7 cells and BMDMs (n = 6). (**C**) The mRNA expression of VEGFA in RAW264.7 cells and BMDMs (n = 3). (**D**) The mRNA expression of IL-1β and TNF-α in kidneys (n = 3). The data are presented as the mean ± SD, * *p* < 0.05 compared with the CON group. ^#^ *p* < 0.05 compared with the ALD group. Scale bar = 200 µm.

We extracted protein and mRNA from RAW264.7 cells and bone marrow-derived monocytes/macrophages (BMDMs) treated with aldosterone and esaxerenone and found that the expression of VEGFA in the ALD group was upregulated, and this effect was reversed in the ESA group (Figure 5B,C). The results of F4/80 and VEGFA costaining in RAW264.7 cells treated with aldosterone and esaxerenone in vitro were similar to the results in vivo (Figure 5A). Furthermore, the quantitative real-time PCR results revealed significantly increased levels of IL-1β and TNF-α in aldosterone-treated RAW264.7 cells, and these changes were strongly decreased after esaxerenone treatment (Figure 5D).

**Figure 6:**
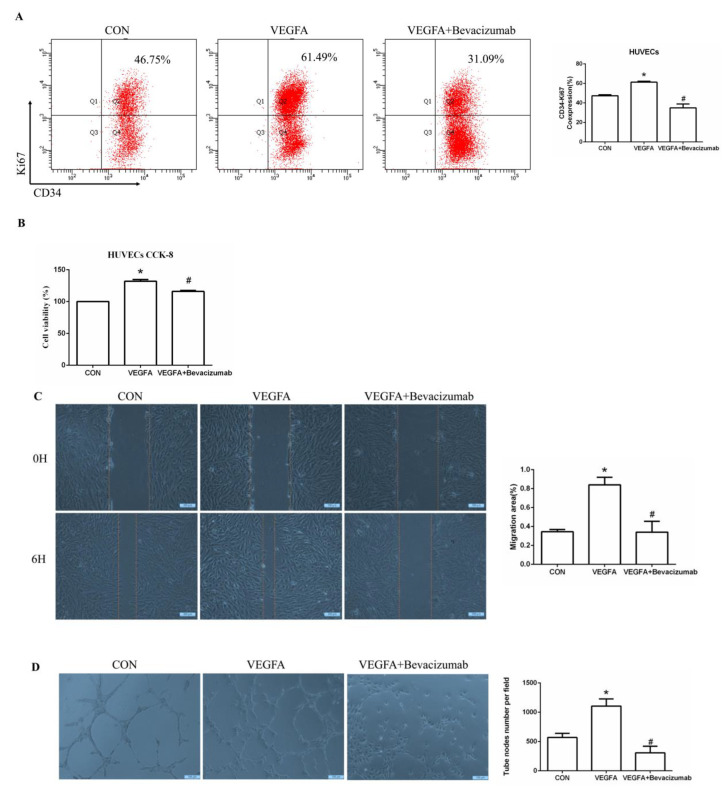

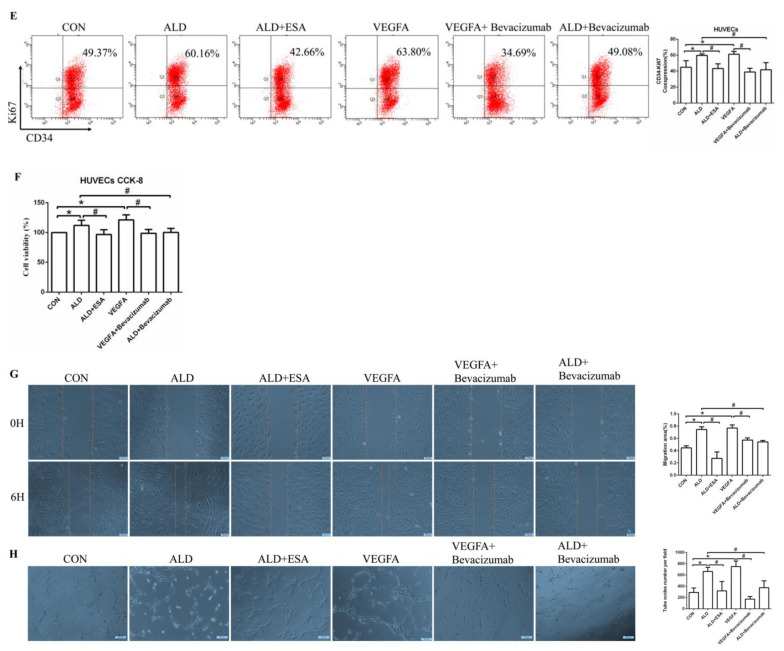
VEGFA promoted angiogenesis by regulating the proliferation, migration, and tube formation of vascular endothelial cells. (**A**) Flow cytometric analysis of the expression of Ki67 in HUVECs, and Q2 shows the percentage of proliferating cells that were Ki67^+^ and CD34^+^ (n = 3). (**B**) CCK8 assays measured VEGFA-induced proliferation in HUVECs (n = 6). (**C**) VEGFA enhanced the migration of HUVECs (n = 3). (**D**) VEGFA enhanced tube formation by HUVECs (n = 3). (**E**) Flow cytometric analysis of the expression of Ki67 in HUVECs, and Q2 shows the percentage of proliferating cells that were Ki67^+^ and CD34^+^ (n = 3). (**F**) CCK8 assays measured VEGFA-induced proliferation in HUVECs (n = 6). (**G**) VEGFA enhanced the migration of HUVECs (n = 3). (**H**) VEGFA enhanced tube formation by HUVECs (n = 3). The data are presented as the mean ± SD, * *p* < 0.05 compared with the CON group. ^#^ *p* < 0.05 compared with the ALD group or the VEGFA group.

To further determine whether VEGFA plays an important role in angiogenesis, we cultured human umbilical vein endothelial cells (HUVECs) with VEGFA (50 ng/mL) for 24 h and found that the expression of the nuclear proliferation marker Ki67 was upregulated, as shown by two-color flow cytometry (Figure 6A). The addition of the VEGFA blocker bevacizumab inhibited the expression of Ki67 (Figure 6A). Similarly, we used Cell Counting Kit-8 (CCK8) assays to detect cell proliferation and found that the proliferation of HUVECs in the VEGFA group was significantly higher than that in the CON group (Figure 6B). In addition, as shown in Figure 6C,D, VEGFA directly promoted the migration rate and lumen formation of HUVECs compared with those in the CON group, while bevacizumab and esaxerenone attenuated these effects.

Moreover, the proliferation of HUVECs in the ALD group was increased, and in the ALD + Bevacizumab group, it was lower than that in the ALD group, which indicated that the VEGFA inhibitor could antagonize aldosterone-induced cell proliferation (Figure 6E,F). The cell migration rate and lumen formation in the ALD group were higher than those in the CON group, and those in the ALD + Bevacizumab group were lower than those in the ALD group, which indicated that the VEGFA inhibitor could decrease the aldosterone-induced cell migration rate and lumen formation (Figure 6G,H). In addition, the results of migration and tube formation of HUVECs in the VEGFA + ESA group were not significantly different from those in the VEGFA group (Figure S1A,B). These results suggest that the MR/VEGFA pathway is involved in angiogenesis and plays a regulatory role.

### 2.6. The MR/TGF-β1 Pathway Is Involved in EndMT In Vitro

**Figure 7:**
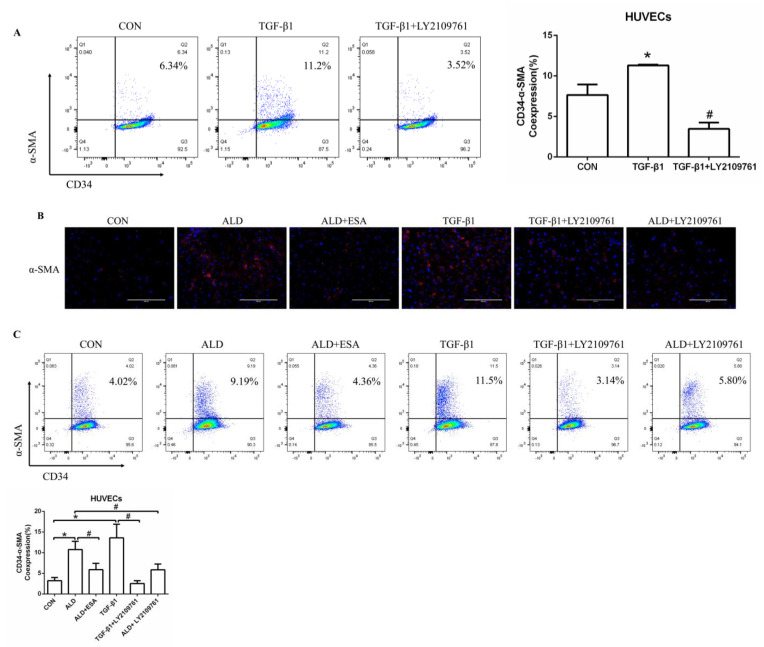

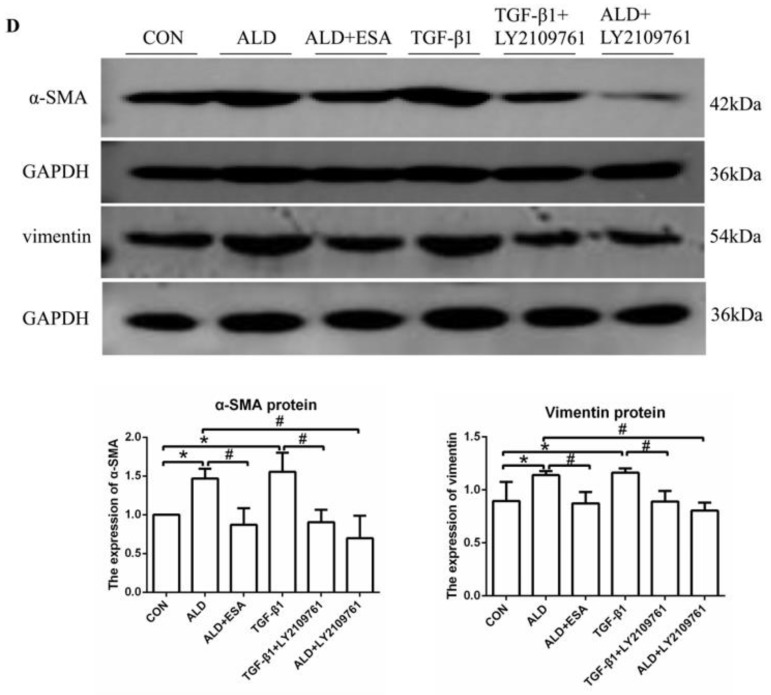
The MR/TGF-β1 pathway is involved in EndMT in vitro. (**A**) Flow cytometric analysis of the expression of α-SMA in HUVECs, and Q2 indicates the percentage of EndMT cells that were α-SMA^+^ and CD34^+^ (n = 3). (**B**) Immunofluorescence staining of HUVECs with antibodies against α-SMA (TRITC, red) to identify EndMT. Nuclei were stained with DAPI (blue). (**C**) Flow cytometric analysis of the expression of α-SMA in HUVECs, and Q2 indicates the percentage of EndMT cells that were α-SMA^+^ and CD34^+^ (n = 3). (**D**) Western blot analysis of α-SMA and vimentin in HUVECs (n = 3). The data are presented as the mean ± SD, * *p* < 0.05 compared with the CON group. ^#^ *p* < 0.05 compared with the ALD group or the TGF-β1 group. Scale bar = 200 µm.

We found that the expression of TGF-β1 in the ALD group of HUVECs and RAW264.7 cells was increased in vitro, which is consistent with our findings in vivo (Figure S1C,D). To further investigate whether TGF-β1 plays an important role in EndMT, we cultured HUVECs with TGF-β1 (10 ng/mL) for 24 h and found that the expression of α-SMA was upregulated (Figure 7A). The addition of the TGF-β1 receptor blocker LY2109761 inhibited the transition of vascular endothelial cells into myofibroblasts (Figure 7A). Moreover, the expression of α-SMA and vimentin in the ALD group was higher than that in the CON group, and the expression in the ALD + LY2109761 group was lower than that in the ALD group, which indicated that the TGF-β1 inhibitor could antagonize aldosterone-induced EndMT (Figure 7B–D). These results suggest that the MR/TGF-β1 pathway is involved in EndMT and plays a regulatory role.

## 3. Discussion

Angiogenesis is a complex process of germination and formation of new blood vessels from preexisting microvessels through migration, proliferation, and survival. Neonatal blood vessels participate in physiological processes, such as development and damage repair, and are also key factors in the pathological process of many diseases, such as cancer [11]. In clinical practice, angiotensin inhibition has been suggested to maintain residual kidney function [12]. Appropriate amounts of VEGFA are beneficial for maintaining glomerular structure, while excessive VEGFA leads to abnormal angiogenesis and is involved in renal fibrosis in diabetic nephropathy [13]. In addition, transgenic rabbits overexpressing VEGFA in the kidney showed significant glomerular damage after 20 weeks and glomerulosclerosis and renal fibrosis after 55 weeks [14]. Interestingly, we detected angiogenesis in the kidneys of the aldosterone-infused mice. The results showed that the renal interstitial endothelial cell marker CD34 and neovascular endothelial cell marker CD105 were abundant in the ALD group, indicating an increase in vascular endothelial cells.

However, angiogenesis can have a protective effect on organs, which has also attracted attention. Subcutaneous VEGFA injection has been shown to preserve peritubular capillary density, prevent interstitial fibrosis, and inhibit the phenotypic transition of endothelial cells to fibroblasts after renal ischemia–reperfusion injury in rodent models [15,16]. EphrinB2/EphB4 is a new pathway associated with angiogenesis that helps treat ischemic cardiovascular disease [17]. Tissue repair after myocardial infarction (MI) involves a robust angiogenic response to improve heart function after MI [18]. Supporting angiogenesis is generally regarded as an effective approach for treating ischemic heart disease, and VEGFA promotes angiogenesis after acute MI by increasing reactive oxygen species (ROS) production and enhancing endoplasmic reticulum (ER) stress-mediated autophagy [19]. Whether the effect of angiogenesis on an organ is beneficial or detrimental depends on the disease and stage. Angiogenesis at sites of acute and subacute injury can repair injury by improving tissue ischemia and hypoxia. However, in the current study, angiogenesis was induced in chronic inflammatory conditions. Angiogenesis might be an aggravating factor for organ fibrosis or a compensatory response observed in acute inflammatory conditions. Specifically, the protective effect of angiogenesis may be a compensatory response that is observed under short-term conditions. Since most neovascularization in fibrotic organs is considered immature and ineffective, it does not improve the ischemic or hypoxic state of the tissue. Conversely, pathological angiogenesis under inflammatory conditions ultimately exacerbates fibrosis [20].

Angiogenesis is related to hypoxia and inflammation. Inflammatory mediators can stimulate angiogenesis, especially in the inflammatory state. Reportedly, IL-1 family cytokines regulate vascular permeability and angiogenesis [21]. Compared with wild-type mice, IL-1β gene knockout mice had significantly reduced neovascularization after ischemic injury [22]. TNF-α induces the proliferation of HUVECs and the formation of new blood vessels, which play key roles in cancer and rheumatic disease [23], and blocking TNF-α can significantly inhibit angiogenesis, tumor growth, and metastasis in vivo [24]. Similarly, in our study, the expression of inflammatory mediators was positively correlated with the degree of angiogenesis, indicating that inflammation induces angiogenesis during the development of CKD.

VEGF is the most important angiogenic factor and mainly includes VEGFA and VEGFC in the kidney. VEGFA plays a key role in angiogenesis [25]. VEGFR2 is the main activating receptor of VEGF, which can be expressed in the cytoplasm of cardiomyocytes, monocytes, smooth muscle cells of the vessel wall, and vascular endothelial cells, indicating the degree of angiogenesis [10]. VEGFA regulates vascular permeability by interacting with VEGFR2 and p-VEGFR2 [26]. Targeted inhibition of VEGFA prevents breast cancer invasion and angiogenesis [27]. Conversely, angiogenesis in colorectal cancer can be directly promoted by upregulating VEGFA expression [28]. Angiogenesis includes several steps that enable different functions of endothelial cells, such as proliferation, migration, lumen formation, differentiation, and maturation [11]. In vitro, VEGFA stimulated HUVECs to increase the expression of Ki67, while bevacizumab, a VEGFA receptor blocker, decreased the expression of Ki67, indicating that VEGFA promoted the proliferation of HUVECs. Furthermore, VEGFA directly promoted cell migration and lumen formation in HUVECs, indicating that VEGFA promoted angiogenesis during the development of CKD.

Many studies have consistently shown that aldosterone induces inflammation and the production of inflammatory mediators that recruit immune cells, such as macrophages and T cells, which are activated by aldosterone and contribute to end-organ damage in cardiovascular and metabolic diseases [29,30]. Macrophages play an important role in kidney injury, inflammation, and fibrosis [31]. They are pleiotropic inflammatory cells that participate in inflammatory reactions, present antigens, and produce cytokines during activation, such as IL-1β and TNF-α [32]. In our study, aldosterone increased the infiltration of inflammatory macrophages in the renal interstitium, and the expression of IL-1β and TNF-α was significantly increased in the ALD group; after aldosterone was blocked with esaxerenone, the infiltration of inflammatory cells and the secretion of inflammatory mediators were decreased. Therefore, we hypothesized that aldosterone induced angiogenesis in an inflammatory environment. Studies have shown that both macrophages and renal tubular epithelial cells can secrete VEGFA [8,9], and the recruitment and significant accumulation of macrophages is an important source of VEGFA during angiogenesis [33,34,35]. Our experiments showed that the aldosterone-treated mice were in an inflammatory state, and aldosterone induced macrophages to secrete high levels of VEGFA to participate in angiogenesis, which was consistent with our finding of abundant macrophage infiltration in the renal interstitium; moreover, in vitro experiments of RAW264.7 cells treated with aldosterone confirmed the discovery in vivo. However, the expression of VEGFA and its effect on angiogenesis after macrophage depletion need to be further investigated.

In addition, studies have shown that hypoxia induces macrophages to secrete VEGFA [33,34]. The formation of a hypoxic environment is related to the overexpression of Na^+^- Cl^−^ cotransporter (NCC), and the enhanced expression of NCC causes water and sodium retention, which, in turn, causes tissue ischemia and hypoxia [36,37]. We used Western blotting and immunofluorescence analysis to show that the expression of NCC, p-NCC (Ser71), p-NCC (Thr53), and hypoxia inducible factor 1 (HIF-1α) was increased in the aldosterone-treated mice (Figure S1E,F, suggesting that aldosterone-induced hypoxia may be involved in macrophage secretion of VEGFA, but this issue will not be discussed further in this study.

Myofibroblasts secrete many extracellular matrix (ECM) proteins, such as collagens and fibronectin, which contribute to renal interstitial fibrosis [38,39,40]. The origin of myofibroblasts in renal fibrosis is controversial, and several cellular sources, including bone marrow-derived fibroblasts, tubular epithelial cells, endothelial cells, pericytes, and interstitial fibroblasts, have been identified [39]. EndMT, which is characterized by the loss of the endothelial phenotype and the acquisition of myofibroblastic properties, also contributes to the deposition of ECM [41,42]. In our study, we found more CD34^+^/α-SMA^+^ cells and CD105^+^/α-SMA^+^ cells in the ALD group than in the CON group, as well as collagen III deposition. In addition, the coexpression of CD34 and α-SMA was detected in vitro by immunofluorescence analysis and flow cytometry, and the results were the same as those in vivo. Therefore, we speculate that EndMT also occurs in neovascular endothelial cells to participate in the pathophysiological process of renal interstitial fibrosis.

As a downstream substrate of MR, TGF-β1 can rapidly induce profibrotic effects through mRNA and protein expression [43]. TGF-β1 induced endothelial cells to undergo EndMT and exacerbated the progression of cardiac fibrosis in a mouse model [42]. In addition, TGF-β1 significantly promoted the development of EndMT in a time- and dose-dependent manner, promoted the motility and migration of HUVECs, and, in turn, exacerbated renal interstitial fibrosis [44]. Our results showed that aldosterone activated MR and then increased the expression of TGF-β1, which indicated that aldosterone may induce EndMT and participate in renal fibrosis through the TGF-β1 signaling pathway. However, whether there are other pathways involved in aldosterone-induced EndMT requires further investigation.

In previous studies, we observed that macrophages in the kidney or heart were involved in fibrosis via macrophage-to-myofibroblast transition (MMT) in UUO rats and aldosterone-infused mice, which was associated with inflammatory injury induced by aldosterone-stimulated MR activation [45,46,47]. MR activation leads to macrophage infiltration and MMT, which participates in fibrosis by secreting the extracellular matrix. The basis of this study is that aldosterone induces macrophage infiltration and secretion of VEGFA and TGF-β1, VEGFA promotes angiogenesis, and TGF-β1 promotes EndMT and secretes collagen III to participate in renal fibrosis. Increased macrophage infiltration is a hallmark of inflammation, the pro-inflammatory mediators secreted by M1 macrophages and the pro-fibrotic factors secreted by M2 macrophages are involved in MMT, angiogenesis, and EndMT. MRB treatment could inhibit MR activation, relieves macrophage infiltration to reduce the release of VEGFA and TGF-β1, etc., could inhibit abnormal angiogenesis and MMT, and could alleviate renal fibrosis. The occurrence and development of MMT and angiogenesis require more in-depth study.

## 4. Materials and Methods

### 4.1. Animals and Experimental Models

All the animal experiments in the research obtained the authorization of the Ethics Committee on Animal Experimentation of the Hebei University of Chinese Medicine and were carried out according to the Care and Use of Laboratory Animals in the National Institutes of Health Guidelines. All efforts were made to minimize pain and distress to the animals. All eight-week-old male (24.7 ± 1.1 g) SPF C57BL/6 mice were provided by the Liaoning Changsheng Biotechnology Co., Ltd., in Changchun, China. In our study we made efforts to provide a comfortable feeling for the experimental mice by applying, for example, suitable room temperature, standard mouse chow and tap water, spacious room, and a 12 h light/12 h dark cycle.

Thirty mice were randomly assigned to the CON group, ALD group (aldosterone group), and ESA group (aldosterone + esaxerenone group) (n = 10 each). Aldosterone is infused with a mini-osmotic pump (0.75 mg/h, ALZET model 2006, DURECT Corporation, Cupertino, CA, USA) as described in a previous paper [47]. Esaxerenone (kindly provided by Daiichi Sankyo Co., Ltd., Tokyo, Japan) was administered to the ESA group via diet at a dose of 1 mg/kg diet for 12 weeks, and the other groups were fed regular chow. Twelve weeks after surgery, the animals were euthanized, and blood and kidney tissue samples were collected for subsequent analysis.

### 4.2. Blood Pressure and Biochemical Parameter Analysis

Systolic blood pressure (SBP) was measured weekly by the tail-cuff method (BP-2000, Visitech Systems, Apex, NC, USA) to monitor changes in blood pressure. Urine samples were collected before the end of the experiment to measure UCr and mAlb levels. UCr was measured by commercial kits (Beckman Coulter Experiment System Co., Ltd., Suzhou, China, No. AUZ3562). mAlb was measured by commercial kits (Nanjing Jiancheng Bioengineering Institute, Nanjing, China, No. E038-1-1). From these, the ACR was calculated.

### 4.3. Histological Analysis, Immunohistochemistry, and Immunofluorescence Analysis

Fresh kidney tissues were fixed in 4% paraformaldehyde (PFA), embedded in paraffin blocks, rendered into 6 μm thick sections, and stained with H&E, Masson, and immunohistochemistry for CD34 (1:100, Abcam, Cat#: ab81289, Cambridge, UK), CD105 (1:100, Abcam, Cat#: ab230925), F4/80 (1:200, Servicebio, Cat#: GB113373, Wuhan, China), CD68 (1:200, Abcam, Cat#: ab283654), α-SMA (1:200, Abcam, Cat#: ab202509), vimentin (1:200, Abcam, Cat#: ab8978), VEGFA (1:100, Abcam, Cat#: ab1316), VEGFR2 (1:100, Abcam, Cat#: ab221679), IL-1β (1:100, Abcam, Cat#: ab283822), and TNF-α (1:100, Abclonal, Cat#: A0277, Woburn, MA, USA). The results were observed and analyzed using a Leica BX53 optical microscope (Leica, Wetzlar, Germany).

H&E staining was used to determine histopathological changes, and the identification of inflammatory cell infiltration and tubulointerstitial changes in the figure was semiquantitatively graded by two investigators in a blinded fashion. Scores include 0, 1, 2, and 3 (normal, mild, moderate, and severe, respectively), on a total score of 0–6 [48]. Determination of the percentage of the collagen-positive area in Masson staining for semiquantitative analysis of the degree of fibrosis. ImageJ software V1.52 (National Institutes of Health, Bethesda, MD, USA) was used for image analysis.

For immunofluorescent staining, the kidneys were fixed with 4% PFA and dehydrated in 30% sucrose. Dehydrated tissues were embedded with OCT compound (Sakura, Torrance, CA, USA) and frozen, and sliced (thickness 6 µm) using a cryostat. After antigen retrieval and blocking with goat serum, the sections were incubated with Alexa Fluor 555-conjugated α-SMA (1:500, Abcam, Cat#: ab202509) or the following unconjugated antibodies: anti-collagen III (1:100, Abcam, Cat#: ab283694), anti-CD34 (1:100, Abcam, Cat#: ab81289), anti-CD105 (1:100, Abcam, Cat#: ab230925), anti-F4/80 (1:200, Servicebio, Cat#: GB113373), anti-VEGFA (1:100, Abcam, Cat#: ab1316), and anti-NCC (1:100, ab95302, Abcam) overnight at 4 °C. Further, a second or third fluorescent staining was performed on the sections. After staining, the sections were incubated with or without DAPI for nuclear staining and observed using confocal microscope (CTS SP8, Leica, Germany).

### 4.4. Protein Extraction and Western Blot Analysis

Protein was extracted from the kidneys (1040μg), RAW264.7 cells, BMDMs, and HUVECs. After electrophoresis, membrane transfer, and 5% nonfat milk incubation, the PVDF membranes were incubated with primary antibodies against α-SMA (1:50, Abcam, Cat#: ab270993), vimentin (1:50, Abcam, Cat#: ab8978), VEGFA (1:50, Abcam, Cat#: ab1316), VEGFR2 (1:50, Abcam, Cat#: ab221679), IL-1β (1:50, Abcam, Cat#: ab283822), TNF-α (1:50, Abclonal, Cat#: A0277), TGF-β1 (1:50, Abcam, Cat#: ab215715), NCC (1:100, ab95302, Abcam), p-NCC (Ser71) (1:1000, Hebei Biohigh Technology Deve Co., LID, China), and p-NCC (Thr53) (1:1000, Hebei Biohigh Technology Deve Co., LID, Shijiazhuang, China) overnight at 4 °C. Then, the membranes were incubated with fluorescein-conjugated secondary antibodies for 1 h at room temperature and scanned with e-BLOT (Shanghai e-BLOT Photoelectrics Technology Co., Ltd., Shanghai, China) or Odyssey (LICOR, Lincoln, NE, USA). ImageJ was used to quantify the density of the target protein relative to GAPDH (1:1000, Proteintech, Cat#: 60004-1-lg, Wuhan, China) and β-Tubulin (1:1000, Affinity, Cat#: T0023, Changzhou, China).

### 4.5. Reverse Transcription and Quantitative Real-Time PCR

The gene expression levels of VEGFA, IL-1β, TNF-α, and GAPDH were measured using real-time PCR in the RAW264.7 cells and BMDMs. Total RNA was extracted as described in a previous paper [47]. Primer sequences were as follows: forward 5′-GTAACGATGAAGCCCTGGAGTG-3′, reverse 5′-TCACAGTGAACGCTCCAGGAT-3′; IL-1β: forward 5′-CTACAGGCTCCGAGATGAACAA-3′, reverse 5′-TTCTTCTTTGGGTATTGCTTGG-3′; TNF-α: forward 5′-CTCTTCTGTCTACTGAACTTCGGG-3′, reverse 5′-GGTGGTTTGTGAGTGTGAGGGT-3′; and GAPDH: forward 5′-CCTCGTCCCGTAGACAAAATG-3′, reverse 5′-TGAGGTCAATGAAGGGGTCGT-3′. VEGFA, IL-1β, TNF-α, and GAPDH were used as housekeeping genes for normalization, and mRNA expression was calculated using the 2^−ΔΔCT^ method.

### 4.6. In Vitro Cell Culture Assays

The source and culture of RAW264.7 cells and fresh bone marrow cells (BMDMs) were the same as described in a previous paper [47]. HUVECs (Sciencell, Carlsbad, CA, USA) were cultured in endothelial cell medium (ECM, Sciencell, Carlsbad, CA, USA) containing 10% FBS, 1% endothelial cell growth supplement (ECGS), 1% penicillin and streptomycin, and maintained in a humidified atmosphere with 5% CO_2_ at 37 °C. After the density of the HUVECs reached 80%, the cells were incubated in serum-free medium for 24 h prior to the cell experiments.

RAW264.7 cells, BMDMs, and HUVECs were divided into the CON group, ALD group (10^−7^ mol/L aldosterone was administered for 24 h), and ESA group (cells were pretreated with 10^−6^ mol/L esaxerenone 2 h prior to aldosterone treatment). In some experiments, HUVECs were induced with 50 ng/mL VEGFA (Proteintech, Wuhan, China, Cat#: HZ-1038) and treated with or without the VEGFA receptor blocker bevacizumab (4 μg/mL) (MCE, Shanghai, China, Cat#: HY-P9906), and HUVECs were treated with bevacizumab after aldosterone induction. HUVECs were induced with 10 ng/mL TGF-β1 (MCE, Shanghai, China, Cat#: HY-P70543) and treated with or without the TGF-β1 receptor blocker LY2109761 (2 × 10^−6^ mol/L) (MCE, Shanghai, China, Cat#: HY-12075), and HUVECs were treated with LY2109761 after aldosterone induction.

#### 4.6.1. Flow Cytometry

Twenty-four hours after treatment, HUVECs were harvested and stained with fluorescein isothiocyanate (FITC)-conjugated anti-CD34 (1:100, Biolegend, Cat#:343504, San Diego, CA, USA), APC-conjugated anti-α-SMA (1:1000, Abcam, Cat#: ab202296), and FITC-conjugated anti-Ki67 (1:100, Invitrogen, Cat#: 11569882, Waltham, MA, USA). Cells were analyzed by BD FACSAria II flow cytometer (BD Biosciences, Franklin Lake, NJ, USA), viable singlet cells were selected by FSC/SSC gating, and results were further analyzed by FlowJo 10 software.

#### 4.6.2. CCK8 Assay

Performed the CCK8 (MCE, Shanghai, China, Cat#: HY-K0301) assay, according to the manufacturer’s instructions. HUVECs were divided into the CON, VEGFA, and VEGFA + Bevacizumab groups and seeded at a density of 10^6^ cells/well in a 96-well plate. Twenty-four hours after stimulation, added 10 μL CCK8 solution to each well and incubated for 2 h. AVersaMax Microplate reader (Molecular Devices, Sunnyvale, CA, USA) was used to determine the absorbance at 470 nm. 

#### 4.6.3. Immunofluorescence Cell Staining

The cells were cultured on 24-well chamber slides and fixed with 4% paraformaldehyde for 20 min at 4 °C. After 0.25% Triton X-100 was added and incubated for 15 min for permeabilization, the cells were incubated with antibodies against CD34, α-SMA, F4/80, and VEGFA overnight at 4 °C. Then, the cells were incubated with TRITC/FITC-labeled secondary antibodies (1:200) for 1 h at 37 °C and the nuclei were stained with DAPI for 10 min. Images were captured using a confocal microscope (CTS SP8, Leica, Weztlar, Germany) and EVOS^®^ FL Auto (Thermo Fisher, Waltham, MA, USA).

#### 4.6.4. Scratch Wound Assay

HUVECs in 6-well plates were scratched by the tip of a 200-μL pipette and treated with VEGFA with or without the VEGFA receptor blocker bevacizumab, treated with aldosterone with or without the aldosterone receptor blocker esaxerenone, and treated with aldosterone and the VEGFA receptor blocker bevacizumab. After the cells were incubated for an appropriate time, photographs were taken under a microscope.

#### 4.6.5. Endothelial Tube Formation Assay

Matrigel Matrix (BD Biocoat 356234, Corning Inc., Corning, NY, USA) was added to a prechilled 24-well plate (100 μL) and allowed to solidify for 1 h at 37 °C. Cells (1 × 10^5^) that had been treated for 24 h were suspended in 100 μL of growth medium containing 5% FBS and seeded on solidified Matrigel. After 4 h of routine culture, every tube with a connected wall of endothelial bundles was counted as one tube under medium magnification (×10). The mean value of the three areas represents the tube-formation capacity of endothelial cells.

### 4.7. Statistical Analysis

GraphPad Prism 6 (GraphPad Software, Inc., La Jolla, CA, USA) was used for data analysis. The data are represented as the mean ± standard deviation. One-way ANOVA and Student–Newman–Keuls Q tests were used to compare differences between the groups. For all statistical tests, a two-tailed *p* value < 0.05 was considered statistically significant.

## 5. Conclusions

In conclusion, we performed in vitro and in vivo experiments and demonstrated that aldosterone induced inflammatory injury and stimulated macrophages to secrete VEGFA to regulate angiogenesis. Neovascular endothelial cells underwent EndMT in response to TGF-β1 to participate in the development of renal interstitial fibrosis, and the MR blocker esaxerenone antagonized these effects. These findings suggest that targeting MR may be an effective therapeutic strategy for renal fibrosis.

## Data Availability

The raw data supporting the conclusions of this article will be made available by the authors, without undue reservation.

## Supplementary Materials

The following supporting information can be downloaded at: https://www.mdpi.com/article/10.3390/ijms241411766/s1.
