# Supplementary material for: Esaxerenone Inhibits Renal Angiogenesis and Endothelial-Mesenchymal Transition via the VEGFA and TGF-β1 Pathways in Aldosterone-Infused Mice
Source: Int J Mol Sci. 2023 Jul 21;24(14):11766. doi: 10.3390/ijms241411766 (PMC10380380; doi:10.3390/ijms241411766)
Supplement: Supplementary file 1 [file ijms-24-11766-s001.zip › ijms-2471334 Supplementary Figure S1.pdf]

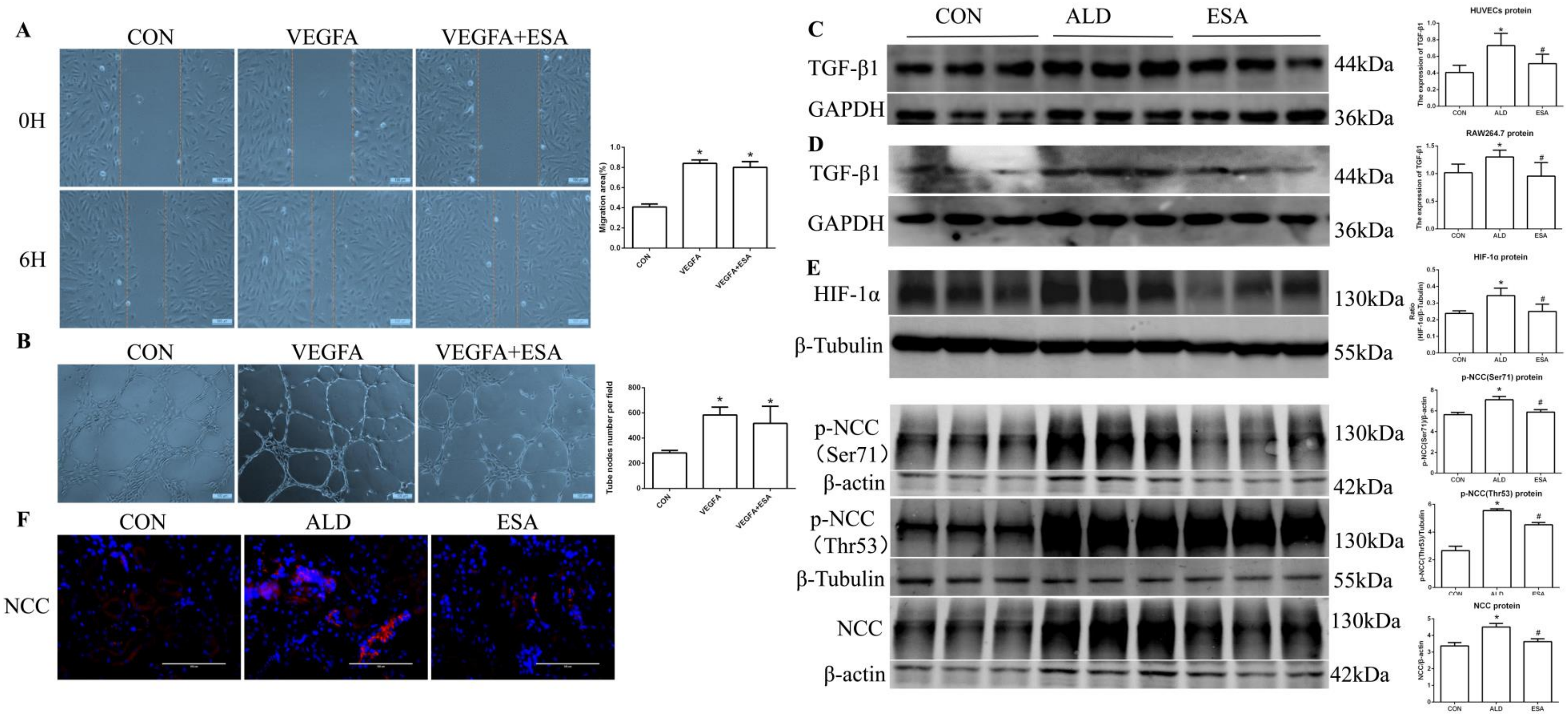

Figure S1. (A) VEGFA+ESA could not enhance the migration of HUVECs (n = 3). (B) VEGFA+ESA could not enhance tube formation of HUVECs (n = 3). (C) Western blot analysis of TGF-β1 in HUVECs (n = 6). (D) Western blot analysis of TGF-β1 in RAW264.7 cells (n = 6). (E) Western blot analysis of NCC, p-NCC (Ser71), p-NCC (Thr53) and HIF-1α in the kidney (n = 3). (F) Immunofluorescence staining of kidney sections with antibodies against NCC (TRITC, red). Nuclei were stained with DAPI (blue). The data are presented as the mean ± SD, * $p < 0.05$ compared with the CON group. # $p < 0.05$ compared with the ALD group
